# Supplementary material for: The Systems Biology Research Tool: evolvable open-source software
Source: BMC Syst Biol. 2008 Jun 29;2:55. doi: 10.1186/1752-0509-2-55 (PMC2446383; doi:10.1186/1752-0509-2-55)
Supplement: Additional file 1 — SBRT Archive. An archive of the current version of the Systems Biology Research Tool. [file 1752-0509-2-55-S1.zip › sbrt-1.4.0/doc/developers_guide/api/sbrt/kernel/math/IntervalComparer.html]

IntervalComparer


|  |  |  |
| --- | --- | --- |
| **Overview** \| **Package** \| **Class** \| **Use** \| **Tree** \| **Deprecated** \| **Index** \| **Help** | |  |
| **PREV CLASS**   **NEXT CLASS** | **FRAMES**    **NO FRAMES**     **All Classes** |  |
| SUMMARY: NESTED \| FIELD \| CONSTR \| METHOD | DETAIL: FIELD \| CONSTR \| METHOD |  |


---


## sbrt.kernel.math Class IntervalComparer

```
java.lang.Object
  sbrt.kernel.math.IntervalComparer
```

---

``` public class IntervalComparer extends java.lang.Object ```

This class is used to compare vectors of intervals.

**Author:**
:   This class was written and documented by Jeremiah Wright while in the
    Wagner lab.

---

| **Nested Class Summary** | |
| --- | --- |
| `static class` | `IntervalComparer.Diff<V>`             Instances of this class are used to hold a variable and a pair of corresponding intervals. |


**Constructor Summary**

`IntervalComparer()`


**Method Summary**

`<V> java.util.Set<IntervalComparer.Diff<V>>`

`getDiffs(java.util.Map<? extends V,Interval> m1,
java.util.Map<? extends V,Interval> m2,
double tolerance)`

**Methods inherited from class java.lang.Object**

`clone, equals, finalize, getClass, hashCode, notify, notifyAll, toString, wait, wait, wait`

**Constructor Detail**

### IntervalComparer

```
public IntervalComparer()
```


**Method Detail**

### getDiffs

```
public <V> java.util.Set<IntervalComparer.Diff<V>> getDiffs(java.util.Map<? extends V,Interval> m1,
                                                            java.util.Map<? extends V,Interval> m2,
                                                            double tolerance)
```


---


|  |  |  |
| --- | --- | --- |
| **Overview** \| **Package** \| **Class** \| **Use** \| **Tree** \| **Deprecated** \| **Index** \| **Help** | |  |
| **PREV CLASS**   **NEXT CLASS** | **FRAMES**    **NO FRAMES**     **All Classes** |  |
| SUMMARY: NESTED \| FIELD \| CONSTR \| METHOD | DETAIL: FIELD \| CONSTR \| METHOD |  |


---
